# Effectiveness of comprehensive rehabilitation for functional recovery and symptom reduction in long COVID: results from a six‑month randomised controlled trial

**DOI:** 10.1038/s41598-026-51787-2

**Published:** 2026-05-05

**Authors:** Katarzyna Anna Pietranis, Anna Kuryliszyn-Moskal, Diana Moskal-Jasińska, Mariusz Wojciuk, Mariusz Ciołkiewicz, Katarzyna Kaniewska

**Affiliations:** 1 Department of Rehabilitation, Medical University of Bialystok, 24A M. Skłodowskiej-Curie St., Bialystok, 15-276 Poland; 2 Department of Clinical Phonoaudiology and Speech Therapy, Medical University of Bialystok, 37 Szpitalna St., Bialystok, 15-295 Poland

**Keywords:** Long-COVID, COVID-19, Post-COVID, Post-COVID rehabilitation, Respiratory muscles training, Respiratory and speech rehabilitation, Diseases, Health care, Medical research, Signs and symptoms

## Abstract

This study provides a comprehensive evaluation of the long-term clinical outcomes of an original rehabilitation programme for patients with long-COVID, highlighting the sustained therapeutic benefits over a six-month period. Seventy‑five patients with long‑COVID symptoms completed a six‑week programme comprising three weekly outpatient sessions. Participants were randomly assigned to two groups following an identical comprehensive protocol, incorporating aerobic training, respiratory exercises, strength and general fitness training, stretching, and respiratory resistance training with a respiratory muscle trainer, with the control group receiving placebo IMT. The study evaluated the programme’s effectiveness by assessing respiratory function (spirometry, muscle strength, chest expansion, and DTF), voice and speech quality, and symptom persistence. The six-month follow-up analysis demonstrated statistically significant retention of improvements in functional and qualitative parameters compared with post-rehabilitation values, with further gains relative to baseline. Our findings indicate that the multi‑component rehabilitation programme is broadly beneficial, with the greatest functional gains in patients with lower baseline capacity, while offering equitable, low‑cost support across age and sex groups and enabling targeted allocation of intensive monitoring to those with the highest potential for clinically meaningful improvement. The study is the first comprehensive analysis of the relationship between long‑COVID and voice and speech‑related disabilities, while also validating the clinical efficacy of the intervention.

**Clinical trials registration**: NCT05449379; 08/07/2022.

## Introduction

Rehabilitation is a fundamental component of the therapeutic approach to pathological changes resulting from COVID-19. A well-structured rehabilitation programme has the potential to enhance exercise capacity, improve balance, muscle function, and pulmonary performance, as well as overall quality of life and psychological well-being^1−6^. Among the various disorders associated with COVID-19, including those observed in post-COVID-19 syndrome, impairments in respiratory function can adversely affect breathing patterns, respiratory-phonatory-articulatory coordination, voice and speech quality, as well as patients’ general functional capacity and their ability to perform everyday tasks^7−10^. A comprehensive rehabilitation protocol not only produces measurable improvements in patient functioning but also enables the early identification of potential health or functional problems that may emerge in later stages of the disease. These include dysfunctions such as dysphonia, sleep disturbances, anxiety, and other mental health disorders^11,12^.

Previous research conducted by our team on the efficacy of rehabilitation in pulmonary arterial hypertension demonstrated that interval aerobic training improves exercise tolerance, alleviates breathlessness and fatigue, optimises breathing patterns, and increases lung volume^13^. Integrating aerobic interval training with breathing exercises strengthens respiratory muscles, enhances chest and diaphragm mobility, and reduces respiratory effort, thereby improving overall respiratory function and boosting physical performance, cardiopulmonary endurance, and muscle strength. These findings provided the foundation for a more extensive investigation into long-term post-COVID-19 complications. Consequently, a randomised study was conducted among patients recovering from COVID-19, demonstrating that the implemented rehabilitation programme is highly effective in improving functional respiratory parameters^14^.

The present study aims to evaluate the long-term effects of an original rehabilitation programme in the same cohort of post-COVID-19 patients who previously participated in that randomised controlled trial.

## Methods

The present study continues an interventional, double-blind, prospective randomised controlled trial (RCT) evaluating the effectiveness of a six-week post-COVID rehabilitation programme. A two-group randomisation was performed using Research Randomizer (Version 4.0). To ensure allocation concealment, the procedure was managed by an independent researcher not involved in patient qualification, data collection, or statistical analysis. Allocation results were stored in secure location and revealed to the clinical team only after the eligibility assessment was completed. To assess the long-term effects of rehabilitation, the intervention and control groups were merged, as no significant differences were observed between them in the investigated parameters except for spirometric values (FEV_1_, FVC, VC_MAX,_ and PEF). A detailed methodology and the short-term effects of rehabilitation on primary parameters assessed in both groups have previously been published^14^. The primary outcomes of this study were functional exercise capacity, measured by the six-minute walk distance (6MWD), and respiratory muscle strength, evaluated via maximal inspiratory pressure (PImax) and maximal expiratory pressure (PEmax). Secondary outcomes included spirometric parameters, chest wall mobility, diaphragmatic ultrasound, and clinical scores for dyspnoea and symptom persistence.

The study is part of a project initiated on 8 July 2022 at the Department of Rehabilitation, Medical University of Bialystok, Poland (ClinicalTrials.gov; NCT05449379; 08/07/2022), examining data collected between August 2022 and April 2024. The research adhered to the ethical principles outlined in the Declaration of Helsinki for medical research involving human participants and received approval from the Bioethics Committee of the Medical University of Bialystok, Poland (APK.002.51.2022). As the previous publication was part of an open project, this long-term analysis incorporates a larger cohort. The study protocol remained unchanged after participant enrollment commenced. Prior to the study, informed consent was obtained from each participant, both verbally and in writing. Patients were fully informed of the study’s objectives, procedures, and potential risks. Data collection occurred in person during diagnostic visits at three time points: (1) pre-rehabilitation eligibility screening, (2) upon completion of the six-week exercise programme, and (3) six months post-rehabilitation.

### Eligibility criteria

The primary inclusion criterion was a confirmed history of COVID-19, verified by RT-PCR testing of nasopharyngeal swabs, with infection occurring within the previous 12 months. Eligibility criteria included systemic post-COVID-19 complications, evaluated using the Post-COVID-19 Functional Status (PCFS) scale (scores of 1–4), and dyspnoea, indicated by a score of ≥ 1 (range: 0–4) on the modified Medical Research Council (mMRC) dyspnoea scale. Exclusion criteria included obesity, gastro-oesophageal reflux disease, and pre-existing chronic respiratory diseases (including chronic obstructive pulmonary disease, asthma, cancer, and tuberculosis). The analysis focused on individuals meeting the WHO definition of long COVID, characterised by persistent or new symptoms occurring three months from the initial infection, lasting at least two months, and not explained by an alternative diagnosis^15^. Importantly, no participants engaged in other rehabilitation programmes between study intervention and the six-month follow-up.

### Intervention

The six-week rehabilitation programme (previously described in detail^14^ was structured around individualised training and included four key components: (1) aerobic training on a cycle ergometer; (2) respiratory and speech rehabilitation exercises; (3) progressive overload resistance training, tailored to each participant’s capabilities; and (4) general fitness exercises and stretching techniques, such as post-isometric relaxation. A respiratory muscle trainer (Philips Respironics Threshold IMT) was used for the resistance training. In the intervention group, the workload was individually prescribed based on each participant’s baseline PImax, starting at 45–55% in Week 1 and progressively increasing to 70–80% by Week 6. In contrast, the control group performed respiratory exercises using the same device under unloaded (sham) conditions, with no resistance applied throughout the 6-week programme. Patient allocation to groups was randomised and conducted by an independent researcher uninvolved in recruitment, data collection, or outcome analysis.

The respiratory and speech rehabilitation component was designed to address breathing disorders and enhance vocal function through a comprehensive set of exercises. These included relaxation and tension-release techniques; diaphragmatic breathing training to establish correct respiratory patterns; and targeted exercises to strengthen the diaphragm, prolong the exhalation phase, and ensure consistent expiratory force. Furthermore, phonation exercises were incorporated to activate vocal resonators and enhance respiratory-phonatory-articulatory coordination.

The intervention consisted of 18 supervised training sessions, (60–90 min each), conducted three times weekly in an outpatient setting. Participants also received individually prescribed home exercises to supplement their rehabilitation programme.

### Procedure

All assessments were conducted in the morning, beginning with evaluations of dyspnoea, fatigue, and post-COVID-19 symptoms. Each diagnostic visit included measurements of pulmonary function parameters (maximal inspiratory and expiratory pressures; spirometry), chest wall mobility, diaphragm ultrasonography, and the six-minute walk test (6MWT), performed according to American Thoracic Society (ATS) guidelines^16^. Outcome measures were divided into primary (6MWD, PImax, and PEmax) and secondary (spirometry, chest mobility, ultrasound, voice parameters, and clinical scales) to facilitate a structured analysis.

Each patient underwent comprehensive speech therapy evaluation, including assessments of respiratory function, voice, and speech quality. Breathing disorders were examined through static and dynamic respiration analyses, focusing on breathing pattern, type of breathing, exhalation strength and uniformity, expiratory phase duration, and coordination between exhalation and phonation. Postural alignment and muscle tension were also systematically evaluated.

Each participant’s breathing pattern was examined and classified as clavicular-costal (apical), diaphragmatic-costal, or abdominal. Evaluation also included analysis of respiratory-phonatory-articulatory coordination and voice and speech intensity, assessed during sustained phonation of the vowel “a” and spontaneous speech. Voice and speech intensity were categorised as piano, mezzoforte or forte. Vocal onset (glottal attack) was evaluated and classified as soft, hard, or breathy. Perceptual (auditory) evaluation of voice characteristics was also performed.

To assess voice quality, Maximum Phonation Time (MPT) was measured in seconds as the average of five samples. Phonation times exceeding 20 s were classified as normal; 10–20 s as reduced; and below 10 s as significantly reduced.

### Bias and study size

To minimise bias, an independent researcher blinded to group allocation conducted eligibility assessments and data collection. The exercise supervisor did not participate in participant selection, data collection, or outcome analysis, ensuring study impartiality. The sample size was determined pragmatically, based on the maximum number of patients meeting the inclusion criteria during the study period^14^. A formal a priori power analysis was not performed due to the limited availability of specific effect size data for combined IMT and exercise protocols in the post-COVID-19 population at the time of the study’s inception. All data were rigorously analysed in accordance with the established study protocol. No outliers were identified, and only complete datasets were included in the final analysis. For isolated missing values (randomly missing data points), median imputation was applied to maintain dataset integrity. To evaluate intervention efficacy under optimal conditions, a per-protocol (PP) analysis was performed. Eligibility for final analysis required attendance at ≥ 12 of 18 training sessions, resulting in a study population with a mean adherence of 0.89.

### Statistical methods

Statistical analyses were conducted using Statistica 13.3 and IBM SPSS Statistics software.

Group comparisons and outcome analyses were performed using descriptive statistics: means and standard deviations, medians with interquartile ranges (IQR), and percentages. Non-parametric tests (Wilcoxon signed-rank, McNemar’s, and Mann-Whitney U) were applied due to data distribution. The *r* coefficient was used as a measure of effect size for the Mann-Whitney U, Wilcoxon signed-rank, and Spearman’s rank correlation tests. For the Mann-Whitney U test, $\:r=\:\frac{Z}{\sqrt{N}}\:,\:$where *Z* is the standardised test statistic and *N* is the total number of observations (sum of both groups for the Mann-Whitney U; sum of paired observations for Wilcoxon signed-rank). Effect sizes were interpreted as small (*r* = 0.10), medium (*r* = 0.30), or large (*r* = 0.50)^17,18^. Categorical variables were analysed using χ^2^ test of independence or Fisher’s exact test. For 6MWT analysis, the Trooster and Enright–Sherrill equations predicted target distances^19,20^. Statistical significance was set at *p* < 0.05.

## Results

**Fig. 1:**
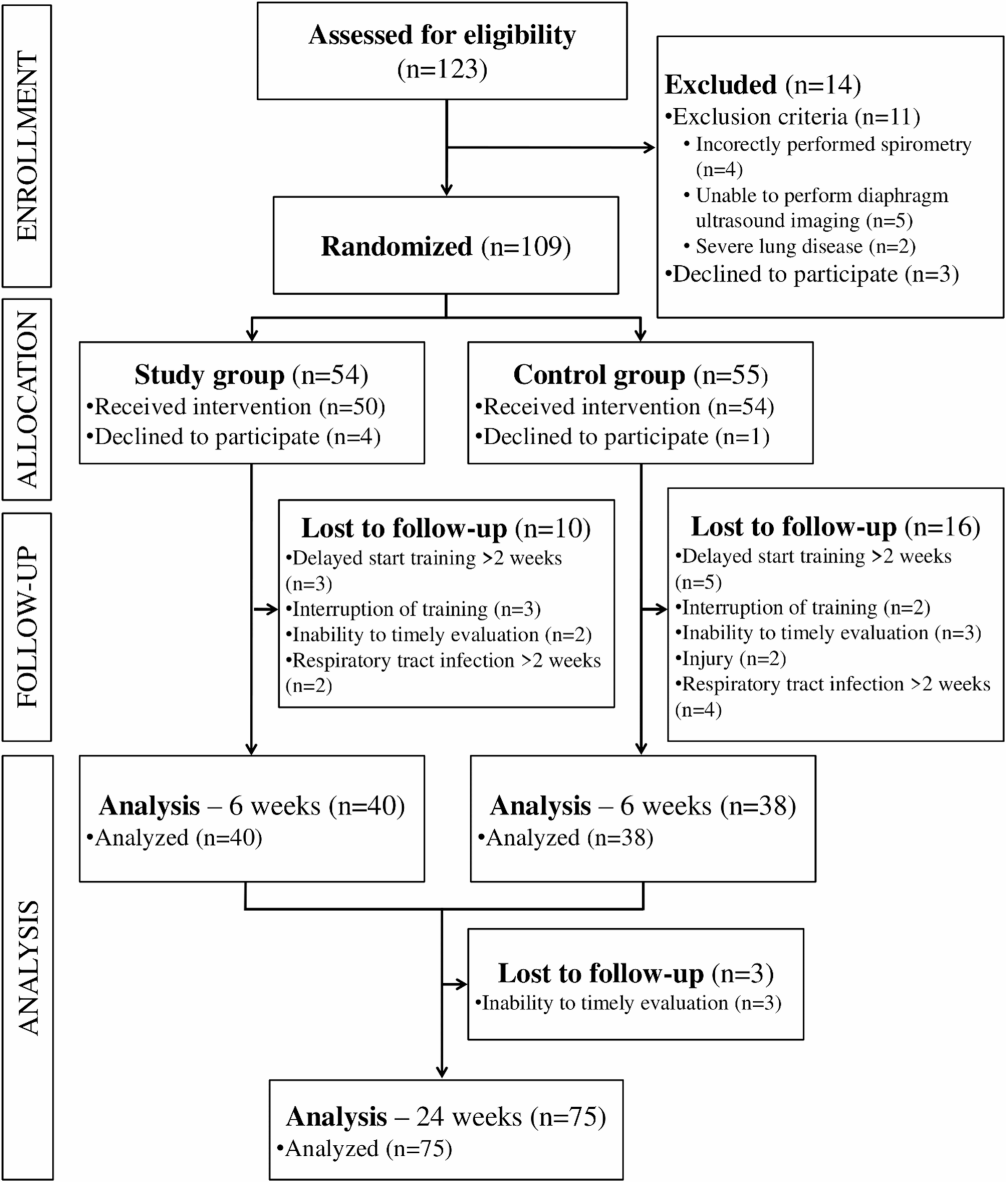
Participant flow diagram.

Of the 123 individuals, 109 were randomised to either the intervention or control group. Figure 1 presents the CONSORT flow diagram illustrating recruitment, allocation, and the follow-up process.

Table 1 summarises a baseline demographic and comorbidity characteristics of participants.

**Table 1:** Baseline participant characteristics before rehabilitation (median [IQR]).

	All patients (*n* = 75)
Age, years	65 [50 − 71]
Male, n (%)	29 (39)
Female, n (%)	46 (61)
BMI, kg/m^2^	28.04 [24.91 − 29.72]
Weight, kg	79.00 [68.00 − 91.50]
Cigarette smoking, n (%)	8 (11)
Hospitalisation during COVID-19, n (%)	14 (19)
Disease onset to examination interval, days	130 [91 − 223]
Hypothyroidism, n (%)	2 (3)
Urinary incontinence, n (%)	3 (4)
Osteoarthritis, n (%)	2 (3)
Hypertension, n (%)	16 (21)
Gastro-oesophageal reflux disease, n (%)	0 (0)

BMI: body mass index.

Tables 2 and 3 show rehabilitation effects at baseline, post-intervention, and six months post- programme. Significant short-term improvements were observed across nearly all assessed parameters. Most gains persisted over 24 weeks post-rehabilitation, with minimal decline from post-intervention to 6-month follow-up. Direct comparison between baseline and 6-month follow-up outcomes confirmed a statistically significant and clinically meaningful impact of the rehabilitation programme.

**Table 2:** Effects of implemented rehabilitation on functional outcomes, pulmonary function and diaphragmatic parameters (median [IQR]).

	Baseline (time 1)	Time 1 → 2		Post-interventional (time 2)	Time 2 → 3		6-month follow-up (time 3)	Time 1 → 3	
		Change	*P*-value		Change	*P*-value		Change	*P*-value
BMI, kg/m^2^	28.04 [24.91 − 29.72]	0 [− 0.34 − 0]	**0.034**	28.04 [25.10 − 29.59]	0 [− 0.32 − 0.35]	0.630	27.55 [24.77 − 29.68]	0 [− 0.39 − 0]	0.197
Functional parameters									
PImax, cmH_2_O	49 [29 − 70]	26 [11 − 41]	**< 0.001**	81 [57 − 98]	1 [− 7 − 11]	0.659	77 [63 − 94]	25 [13 − 41]	**< 0.001**
PEmax, cmH_2_O	63 [53 − 95]	34 [11 − 58]	**< 0.001**	106 [77 − 145]	− 4 [− 29 − 15]	0.134	95 [67 − 129]	23 [7 − 44]	**< 0.001**
6MWD, m	300 [264 − 360]	35 [15 − 85]	**< 0.001**	365 [305 − 400]	15 [− 15 − 55]	**0.02**	368 [330 − 435]	50 [15 − 105]	**< 0.001**
Spirometry, % predicted									
FEV_1_	89 [77 − 101]	9 [4 − 16]	**< 0.001**	100 [90 − 111]	− 1 [− 5 − 3]	0.387	100 [90 − 111]	9 [3 − 16]	**< 0.001**
FVC_EX_	94 [77 − 110]	9 [4 − 20]	**< 0.001**	107 [95 − 122]	0 [− 5 − 4]	0.302	108 [95 − 118]	8 [3 − 22]	**< 0.001**
FVC_IN_	87 [69 − 104]	10.41 [3 − 24]	**< 0.001**	104 [94 − 115]	1 [− 3 − 6]	0.111	106 [94 − 116]	14 [6 − 33]	**< 0.001**
VC_MAX_	93 [78 − 107]	7 [4 − 17]	**< 0.001**	105 [94 − 116]	0 [− 4 − 3]	0.68	106 [93 − 116]	7 [3 – 20]	**< 0.001**
FEV_1_/FVC	74.60 [71.10 − 78.35]	2.05 [0.73 − 3.85]	**< 0.001**	76.21 [72.82 − 81.40]	0.3 [− 1.08 − 1.43]	0.35	76.40 [73.33 − 82.11]	2.4 [0.56 − 4.59]	**< 0.001**
FEV_1_/VC_MAX_	74.53 [71.10 − 78.19]	2.1 [1.06 − 3.57]	**< 0.001**	76.21 [72.83 − 81.00]	0.28 [− 1.53 − 1.42]	0.581	76.26 [73.33 − 80.95]	2.45 [0.56 − 4.22]	**< 0.001**
PEF	87 [73 − 104]	11 [3 − 21]	**< 0.001**	100 [86 − 115]	0 [− 7 − 6]	0.77	100 [89 − 113]	10 [3 − 22]	**< 0.001**
Chest expansion									
Xiphoid process level, cm	2 [1 − 3]	1 [0 − 2]	**< 0.001**	3 [2 − 5]	0.9 [− 0.5 − 2]	**0.001**	4 [3 − 6]	2 [0 − 3.5]	**< 0.001**
10th rib level, cm	2 [1 − 3]	1 [0 − 2]	**< 0.001**	2 [1 − 5]	1 [− 0.5 − 2]	**0.001**	4 [2 − 5]	2 [0.5 − 3]	**< 0.001**
Diaphragm ultrasonography									
DTF	0.931 [0.543 − 1.253]	0.598 [0.319 − 1.089]	**< 0.001**	1.529 [1.037 − 2.384]	0.00 [− 0.302 − 0.334]	0.97	1.639 [1.251 − 2.457]	0.584 [0.197 − 1.341]	**< 0.001**

BMI: body mass index; PI_max_: maximum inspiratory pressure; PE_max_: maximum expiratory pressure; 6MWD: six-minute walk distance; FEV_1_: forced expiratory volume in 1 s; FVC_EX_: forced expiratory vital capacity; FVC_IN_: forced inspiratory vital capacity; VC_MAX_: maximum vital capacity; PEF: peak expiratory flow; DTF: diaphragm thickening fraction − determined by subtracting the diaphragm thickness at end-expiration (DTe) from its thickness at end-inspiration (DTi) and dividing the result by DTe: DTF = (DTi − DTe)/DTe.

Significant values are in bold.

**Table 3:** Effects of implemented rehabilitation on the prevalence of key symptoms (median [IQR]).

	Baseline (time 1)	Time 1 → 2		Post-interventional (time 2)	Time 2 → 3		6-month follow-up (time 3)	Time 1 → 3	
		Change	*P*-value		Change	*P*-value		Change	*P*-value
Dyspnoea (mMRC ≥ 1), n (%)	75 (100)	− 17	**< 0.001**	58 (77)	− 14	**< 0.001**	44 (59)	− 31	**< 0.001**
Dyspnoea (Modified Borg Scale 0–10)	3 [2 − 3]	− 1 [− 2−(− 1)]	**< 0.001**	1 [1 − 2]	0 [− 1 − 2]	**0.027**	1 [0 − 3]	− 1 [− 3 − 1]	**0.02**
RPE Scale (Modified Borg Scale 0–10)	5 [3 − 7]	− 2 [− 4−(− 1)]	**< 0.001**	3 [2 − 4]	0 [− 1 − 1]	0.538	3 [1 − 4]	− 2 [− 4 − 0]	**< 0.001**
Cough, n (%)	55 (73)	− 23	**< 0.001**	32 (43)	11	**0.019**	43 (57)	− 12	**< 0.001**
Headache, n (%)	49 (65)	− 10	**0.002**	39 (52)	4	0.454	43 (57)	− 6	0.21
Chest pain, n (%)	55 (73)	− 12	**< 0.001**	43 (57)	− 10	**0.031**	33 (44)	− 22	**< 0.001**
Muscular or articular pain, n (%)	66 (88)	− 6	**0.041**	60 (80)	− 2	0.773	58 (77)	− 8	**0.013**
Fatigue, n (%)	75 (100)	− 6	**0.031**	69 (92)	− 2	0.5	67 (89)	− 8	**0.008**

RPE: Rate of Perceived Exertion.

Significant values are in bold.

The programme substantially improved functional capacity and respiratory muscle strength, yielding large effect sizes (*r* = 0.614; *p* < 0.001) for PImax and PEmax immediately post-intervention. These gains persisted at 6-month follow-up (*r* = 0.614; *p* < 0.001). Similarly, 6MWD showed large, sustained increases after the intervention (*r* = 0.614; *p* < 0.001) and at 6 months (*r* = 0.615; *p* < 0.001). Spirometry parameters – including FEV_1_, FVC_EX_, FVC_IN_, VC_MAX_, and lung function indices – demonstrated consistently large effect sizes (*r* ≈ 0.61; *p* < 0.001). PEF increased robustly post-intervention (*r* = 0.577) and at follow-up (*r* = 0.615; both *p* < 0.001). Chest expansion exhibited moderate-to-large effect sizes post-rehabilitation, with *r* = 0.398 at the xiphoid process and *r* = 0.417 at the 10th rib level (both *p* < 0.001). Notably, these effects strengthened further by the 6-month follow-up, particularly at the 10th rib level, which achieved a large effect size (*r* = 0.528; *p* < 0.001). Finally, diaphragmatic thickness fraction (DTF) showed consistently large effect sizes throughout the study (*r* ≈ 0.61; *p* < 0.001).

Progressive improvements in 6MWD were observed across all timepoints. This trend was consistent for both absolute distances and predicted values, calculated using the Trooster and Enright– Sherrill equations, which incorporate demographic and anthropometric variables to enhance clinical interpretability^19,20^. At baseline, median predicted 6MWD was 49% [IQR: 43 – 56] and 61% [IQR: 51 – 69] of expected values for the two equations, respectively. Post-rehabilitation, these increased to 56% [IQR: 49 − 63] and 69% [IQR: 60 − 78], respectively. At 6-month follow-up, values rose further to 59% [IQR: 53 − 65] and 73% [IQR: 63 − 80], respectively.

At baseline, three participants scored < 30% of predicted 6MWD using the Enright– Sherrill equation, and eight using the Trooster equation. Only one remained below this threshold at both post-intervention and 6-month follow-up (Trooster equation only). Conversely, ≥ 75% of predicted 6MWD (Enright–Sherrill equation) was achieved by 10 participants at baseline, 27 post-intervention, and 35 at 6-month follow-up. Using the Trooster equation, high results (≥ 75% ) occurred in 5 participants post-intervention and 8 at 6 months. Analysis explored the relationship between the distance traversed and the functional parameters of the respiratory system, revealing positive correlations between the 6MWD and the values of PImax (*r* = 0.397; *p* < 0.001) and PEmax (*r* = 0.252; *p* = 0.029). Greater inspiratory muscle strength was associated with longer 6MWD at 6 months post-rehabilitation. Additionally, distance improvements correlated with greater chest expansion at xiphoid level (*r* = 0.249; *p* = 0.031) and reduced chest pain (*r* = -0.275; *p* = 0.017).

Subgroup analyses were conducted to evaluate the influence of baseline characteristics on functional recovery. Participants stratified by baseline capacity (Enright–Sherrill equation: <75% vs. ≥75% predicted 6MWD) showed significantly different functional gains (*p* = 0.026). Those with lower baseline capacity (< 75%) achieved substantially greater improvements, with a small-to-moderate effect size (*r* = 0.257). Age-stratified analysis (< 65 vs. ≥ 65 years) revealed significantly greater 6MWD gains in younger participants (Z = 2.117, *p* = 0.034; *r* = 0.244), consistent with a weak negative correlation between age and 6MWD change (*r* = -0.262, *p* < 0.05). No significant sex differences were observed in the magnitude of the change in distance (*p* > 0.05).

Sex differences emerged in patient-reported outcomes at 6 months post-rehabilitation. Muscle and joint pain was reported by 56% of women vs. 24% of men (*p* = 0.003). Women were significantly more likely to report headaches (χ^2^ = 7.28; *p* = 0.007) and chest pain (χ^2^ = 5.17; *p* = 0.023). Men achieved significantly greater respiratory muscle strength than women (PImax: *r* = -0.578; *p* < 0.001; PEmax: *r* = -0.538; *p* < 0.001), as well as superior chest expansion at the 10th rib (*r* = -0.241; *p* = 0.037).

Age emerged as a key determinant of treatment outcomes. Increasing age was associated with declines in PImax (*r* = -0.229; *p* = 0.048), PEmax (*r* = -0.231; *p* = 0.046), FEV_1_/FVC (*r* = -0.271; *p* = 0.018) and FEV_1_/VC_MAX_ (*r* = -0.335; *p* = 0.003). Older patients exhibited a lower prevalence of persistent headaches (*r* = -0.255; *p* = 0.027) and covered significantly shorter 6MWD (*r* = -0.451; *p* < 0.001). A moderate positive correlation was established between age and dyspnoea, as assessed by the modified Borg scale (*r* = 0.422; *p* < 0.001), indicating that the perceived severity of breathlessness increased significantly with advancing age. Patients reporting chest pain demonstrated significantly lower PImax (*r* = 0.264; *p* = 0.022), PEmax (*r* = 0.309; *p* = 0.007), and 6MWD (*r* = 0.273; *p* = 0.018). Additionally, the presence of cough was associated with reduced FEV₁ values, showing a small effect size (*r* = 0.23; *p* = 0.046).

Values obtained from the modified Borg scale exhibited a weak negative correlation with diaphragmatic thickening fraction (DTF) (*r* = -0.29; *p* = 0.012). Similarly, weak positive correlations were observed between fatigue and the presence of headaches (*r* = 0.249; *p* = 0.031) and chest pain (*r* = 0.286; *p* = 0.013). Furthermore, musculoskeletal pain demonstrated a moderate positive correlation with fatigue (*r* = 0.306; *p* = 0.008).

In this post-COVID-19 cohort, the most prevalent respiratory symptoms were shortened and shallow breathing, dyspnoea, breathing difficulties, cough, reduced respiratory efficiency, impaired exercise tolerance, and difficulty speaking longer phrases or sentences in a single breath. The most frequently reported phonatory symptoms were hoarseness, vocal fatigue, dryness of the throat mucosa, sore throat, scratchiness, burning sensation, choking, transient voice loss and the sensation of a foreign body in the throat (globus pharyngeus). A summary of changes in breathing patterns, voice parameters and MPT across the three measurement points is presented in Table 4.

**Table 4:** Summary of voice and speech quality parameter assessment.

	Baseline (time 1)	Post-intervention (time 2)	6-month follow-up (time 3)
Breathing patterns			
Clavicular-costal, n (%)	66 (88%)	16 (21%)	18 (24%)
Diaphragmatic-costal, n (%)	8 (11%)	59 (79%)	57 (76%)
Abdominal, n (%)	1 (1%)	–	–
Respiratory-phonatory-articulatory coordination disorders, n (%)	69 (92%)	28 (37%)	32 (43%)
Voice intensity			
Piano, n (%)	40 (53%)	12 (16%)	13 (17%)
Mezzoforte, n (%)	9 (12%)	55 (73%)	51 (68%)
Forte, n (%)	26 (35%)	8 (11%)	11 (15%)
Vocal onset			
Soft, n (%)	12 (16%)	52 (69%)	47 (63%)
Hard, n (%)	29 (39%)	9 (12%)	13 (17%)
Breathy, n (%)	34 (45%)	14 (19%)	15 (20%)
Maximum Phonation Time			
Normal, n (%)	6 (8%)	18 (24%)	16 (21%)
Reduced, n (%)	43 (57%)	44 (59%)	50 (67%)
Significantly reduced, n (%)	26 (35%)	13 (17%)	9 (12%)

## Discussion

The rehabilitation programme demonstrated substantial efficacy in improving functional capacity and respiratory muscle strength in post-COVID-19 patients, with most gains sustained over 6 months. Large effect sizes were consistently observed across key parameters, including PImax, PEmax, 6MWD, spirometry indices (FEV₁, FVC_EX_, FVC_IN_, VC_MAX_, PEF), and diaphragmatic thickness fraction (DTF), all achieving *r* ≈ 0.61 (*p* < 0.001). These findings align with the per-protocol analysis criteria (≥ 12/18 sessions; mean adherence 0.89), confirming intervention effectiveness under optimal compliance.

The six-week rehabilitation programme demonstrated durable efficacy in improving pulmonary function and overall health outcomes in post-COVID-19 patients. No significant health deterioration was observed at 6-month follow-up, confirming the stability and longevity of therapeutic effects. Despite these positive outcomes, marginal reduction in PImax and PEmax values, alongside increased reports of cough and headaches during follow-up, suggest the need for sustained physical activity to maintain health benefits. Nevertheless, improvements in respiratory muscle strength remained clinically substantial. Specifically, the PImax increase observed in our cohort (mean change of approximately 30 ± 22 cmH_2_O) significantly exceeded the established Minimal Clinically Important Difference (MCID) threshold of 18 cmH_2_O^21^ Notably, 48 participants achieved PImax improvements meeting or exceeding the MCID threshold. A similar trend was observed for PEmax, with mean improvement reaching approximately 31 ± 31 cmH_2_O. In clinical interventions, statistically significant and clinically relevant PEmax changes typically range between 10 and 20 cmH_2_O^22–24^. Our study results substantially exceeded this range, with 51 participants achieving improvements greater than 10 cmH_2_O, confirming the rehabilitation programme’s tangible clinical impact. Analysis across the three evaluation periods demonstrates at least six months of sustained benefits, reinforcing the long-term effectiveness of the implemented rehabilitation programme.

The rehabilitation programme significantly improved exercise capacity, as evidenced by increased target distance values. The sustained benefits observed after 6-month follow-up highlight the programme’s long-term therapeutic efficacy. An increase of 25 m in 6MWD represents the MCID in patients with respiratory diseases, including chronic obstructive pulmonary disease, asthma, and interstitial lung disease^25^. In this study, mean improvement reached 78 ± 84 m – more than threefold the MCID. Overall, 52 (69%) of participants exceeded this clinically meaningful 6MWD threshold. Reference values for 6MWT distance in COVID-19 patients − determined using the Enright–Sherrill equation − have been primarily investigated by Hayden et al.^26^. In contrast to their findings of a weak negative correlation between the 6MWD and PImax (*r =* -0.280), our study demonstrated a moderate positive correlation between 6MWD improvements and inspiratory muscle strength gains (*r =* 0.397; *p* < 0,001). These findings suggest that the rehabilitation programme enhances coordination between the respiratory and musculoskeletal systems, thereby directly contributing to improved physical performance in individuals recovering from COVID-19.

Our findings suggest that while the multi-component rehabilitation programme is universally beneficial, its efficacy is modulated by baseline functional status and age. The significantly greater gains observed in patients with a baseline 6MWD below 75% of the predicted value (*p* = 0.026) highlight the role of this intervention as a high-priority therapy. In contrast, for higher-performing participants, the programme may serve primarily in secondary prevention – maintaining functional capacity and preventing the further development of chronic symptoms. The observed correlation between age and recovery magnitude (*r* = -0.262) suggests greater physiological plasticity in younger patients^27^. However, the lack of gender-based differences indicates that the programme’s core components provide equitable benefits across the sex spectrum. From a healthcare management perspective, these insights enable more targeted resource allocation. Since the intervention is inherently low-cost and does not rely on expensive specialised equipment, it can be widely implemented. Simultaneously, our findings ensure that intensive clinical monitoring or supplementary support can be prioritised for patient profiles with the greatest potential for clinically significant improvement.

Studies investigating changes in the functional parameters of the respiratory system have revealed a significant trend toward gradual improvement over time^28,29^. Furthermore, the most extensive three-year observational study found that pulmonary function in post-COVID-19 patients largely returned to control group levels. In the present study, a baseline FEV_1_ value below 70% of predicted was observed in 10 participants (13.3%). Following the rehabilitation programme, this decreased to 3 participants (4%), and further to 2 (2.7%) at the six-month follow-up. A similar trend was observed for FVC_EX_ values, with 10 individuals (13.3%) initially below 70% of predicted, decreasing to 3 (4%) post-rehabilitation. However, patients with long-COVID experienced more frequent dyspnoea, higher reinfection rates, an increased risk of pneumonia following Omicron reinfection, and persistent symptoms including fatigue, muscle weakness, sleep disturbances, palpitations, olfactory dysfunction, reduced appetite, sore throat, and headaches. Additionally, lung computed tomography (CT) abnormalities persisted in this group, indicating a long-term impact on respiratory function^30^.

While improvements were observed shortly after rehabilitation, symptoms such as cough and headache became more prevalent at six months. According to established literature for COPD^31^– a population with similar ventilatory limitations – the MCID for the Borg scale is defined as a reduction of 1 unit. In our study, 32 (43%) participants exceeded this threshold, achieving a reduction of ≥ 2 points. This outcome indicates that the multi-component rehabilitation programme provided robust clinical benefit. Possible causes of persistent cough include chronic epithelial irritation, airway inflammation, reduced lung function, and bronchial hyperresponsiveness^32−34^. Garcia-Azorin et al.^35^ proposed that SARS-CoV-2 infection may trigger a predisposition to migraine or contribute to new daily persistent headache (NDPH), although the underlying pathophysiological mechanisms remain unclear^36,37^. Muscle and joint pain was the second most frequently reported complication following fatigue. Zhai et al.^38^ suggested that skeletal muscle pain in long COVID may result from overlapping pathophysiological mechanisms, including chronic inflammation, oxidative stress, and mitochondrial dysfunction.

The potential causes of diaphragmatic dysfunction in post-COVID-19 patients are multifactorial and may involve several overlapping mechanisms. A previous study has examined the issue in depth^39^. Among the documented causes, mechanical ventilation remains the most frequently reported contributor to diaphragmatic injury^40,41^. Veldman et al.^42^ observed marked improvements in diaphragmatic function over time, with DTF increasing from 1.06 at three months to 1.54 at twelve months, suggesting a prolonged recovery trajectory. Our findings partially align with this hypothesis. However, patients who participated in a structured rehabilitation programme exhibited higher DTF values, suggesting a potential added benefit of rehabilitation in facilitating diaphragmatic recovery. Comparison of traditional breathing exercises with technology-based therapies, such as breathing-assist devices, is clinically significant for determining the most effective strategies to improve respiratory and vocal function in long COVID patients. In the absence of standardised rehabilitation protocols for this population, evaluating the long-term efficacy of various approaches is essential, particularly fto enable early adoption of effective strategies in future viral outbreaks.

In a study conducted by Gacka, 27% of post-COVID-19 patients exhibited respiratory dysfunctions, 35% presented with primary functional impairments, and 13% were diagnosed with voice disorders. A similar distribution of symptoms was observed within our cohort, with patients frequently reporting shallow, short breathing, difficulty sustaining longer phrases within a single breath, dyspnoea, and irregular breathing^43^.

Our findings indicate a significant reduction in MPT among post-COVID-19 patients. Prior to rehabilitation, significantly shortened phonation time was observed in 35% of participants, while 57% demonstrated an overall reduction in MPT. Following rehabilitation, improvements were documented: only 17% of participants continued to exhibit significantly reduced MPT, while 59% showed some degree of reduction. These results are consistent with previous studies reporting notable decreases in MPT, particularly among women^44^. Additionally, shortening of the exhalation phase contributes to respiratory-phonatory coordination disorders, which impair the ability to produce longer utterances^9^.

While meta-analyses have established the short-term benefits of post-COVID-19 rehabilitation^45,46^, data regarding long-term physiological outcomes remain relatively scarce and are often confounded by external factors such as reinfection. Notably, none of the participants in our cohort experienced reinfection during the follow-up period, which allowed for a more robust and precise assessment of the intervention’s durable effects. Previous longitudinal studies, such as the 12-month evaluation of a 27-day inpatient programme, have relied primarily on subjective health assessments^47^. In contrast, our study provides a more comprehensive physiological perspective by integrating objective measurements of respiratory function, diaphragmatic activity, and exercise capacity. This objective approach addresses a critical gap in the literature, moving beyond patient-reported outcomes to document the sustained recovery of the respiratory system.

The sustained improvements observed six months post-rehabilitation may be attributed to several mediating factors, most notably long-term behavioural change. As suggested in the literature, successful pulmonary rehabilitation often acts as a catalyst for increased daily physical activity and improved self-efficacy^48^. It is plausible that participants integrated regular exercise into their daily routines, thereby mitigating the detraining effect typically observed after cessation of structured programmes^49^. Notably, our rehabilitation programme did not formally impose specific lifestyle changes or structured physical activity goals. This suggests that the observed durability of functional gains may stem from a spontaneous increase in activity, enabled by the physiological recovery achieved during the intervention. Furthermore, the reduction in exertional dyspnoea likely encouraged a transition from a sedentary lifestyle to more active habits, which has been shown to be a key moderator in sustaining functional gains in patients with chronic respiratory impairments^50,51^.

The clinical significance of these sustained improvements becomes even more apparent when contrasted with the natural course of post-COVID-19 recovery reported in the literature. For instance, Swedish researchers reported that over 80% of patients hospitalised during the first wave continued to experience symptoms 24 months post-infection^52^, while multicentre data indicated that up to 73% of patients had not fully recovered within two years^53,54^. Despite some improvements in physical health over time, neurocognitive complaints, fatigue, and dyspnoea remained persistent in those cohorts. In contrast, our findings demonstrate that a structured rehabilitation programme can actively accelerate recovery and significantly reduce the frequency of symptoms such as fatigue, musculoskeletal pain, and dyspnoea. This suggests that targeted intervention can effectively mitigate the burden of long COVID, offering a more favourable trajectory than passive observation alone.

## Conclusions

The present study demonstrates that an intensive, multi‑component rehabilitation programme yields substantial and immediate health benefits for patients recovering from COVID‑19. Significant improvements were observed in respiratory function, exercise capacity, and voice and speech quality, alongside enhanced respiratory-phonatory-articulatory coordination. Although certain parameters, such as PImax and PEmax, x exhibited a marginal decline six months after rehabilitation, they remained significantly elevated above baseline. Crucially, the functional gains achieved exceeded established thresholds for clinically meaningful improvement (minimally clinically important difference, MCID), and these effects were maintained at the 6-month follow-up.

These findings suggest that the intervention not only induces immediate physiological recovery but also facilitates long-term behavioural changes that mitigate the detraining effects typically associated with post-COVID-19. Overall, this structured rehabilitation approach offers a more favourable recovery trajectory than the natural course of the disease, providing a low-cost and effective strategy for long-term health restoration.

### Generalisability and limitations

The present study demonstrates the clinical efficacy of the rehabilitation programme, with improvements persisting for at least six months after the intervention. Key strengths of the study include the extended follow-up period, high data completeness, and a comprehensive evaluation of both subjective and objective clinical parameters. Importantly, this is the first report to establish a significant association between long COVID and vocal disability.

Long-term outcomes are essential for evaluating the durability of rehabilitation benefits and for enabling the generalisation of findings. The limited number of available randomised trials examining the long-term effects of rehabilitation on functional respiratory parameters and respiratory-phonatory-articulatory coordination may be partly attributable to methodological challenges. These include individual variability in the course of COVID-19, which may be influenced by SARS-CoV-2 variants, vaccination status, and differential responses to physical activity − factors that are inherently difficult to control.

Future multicentre randomised trials involving diverse cohorts, with detailed analyses of pre-pandemic health status and hospitalisation history, are needed to strengthen the evidence base. Additionally, continued evaluation of long-term efficacy is warranted to better determine the sustainability of rehabilitation outcomes in clinical practice.

## Data Availability

The data supporting the findings of this study are not publicly available due to their sensitive nature but may be obtained from the corresponding author upon reasonable request.
